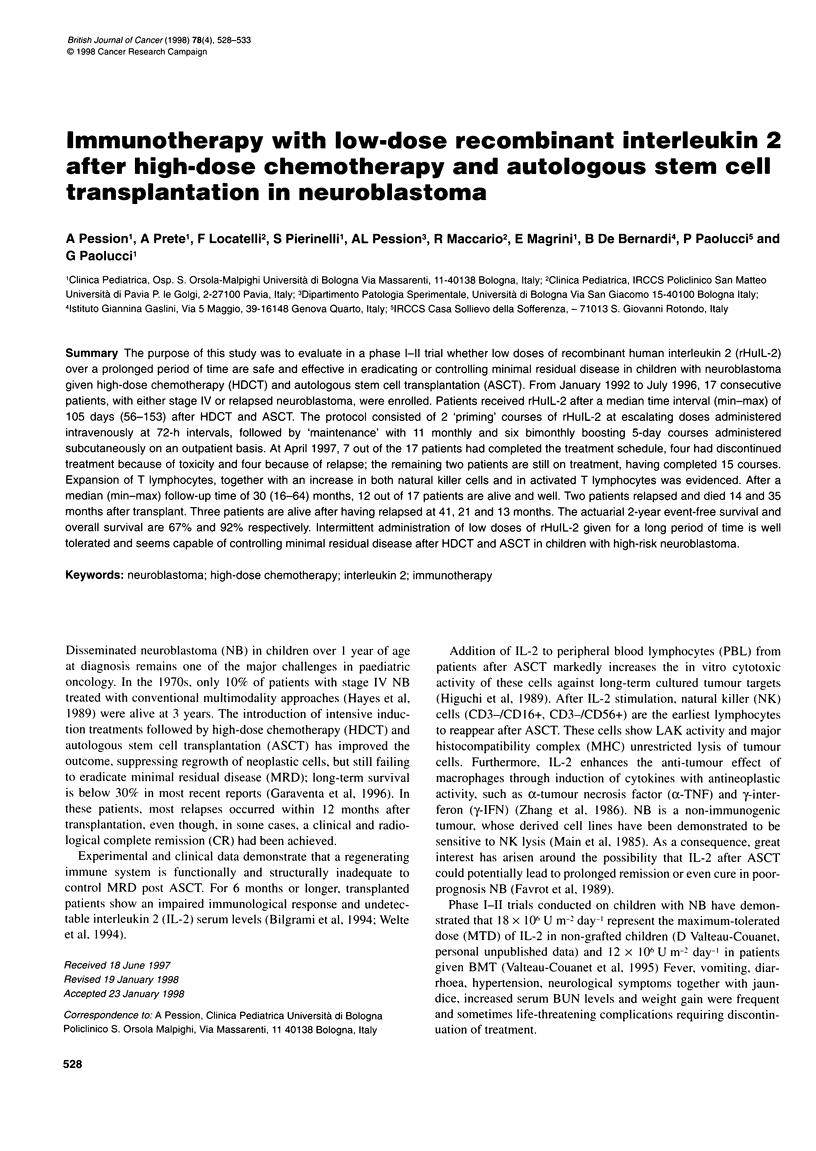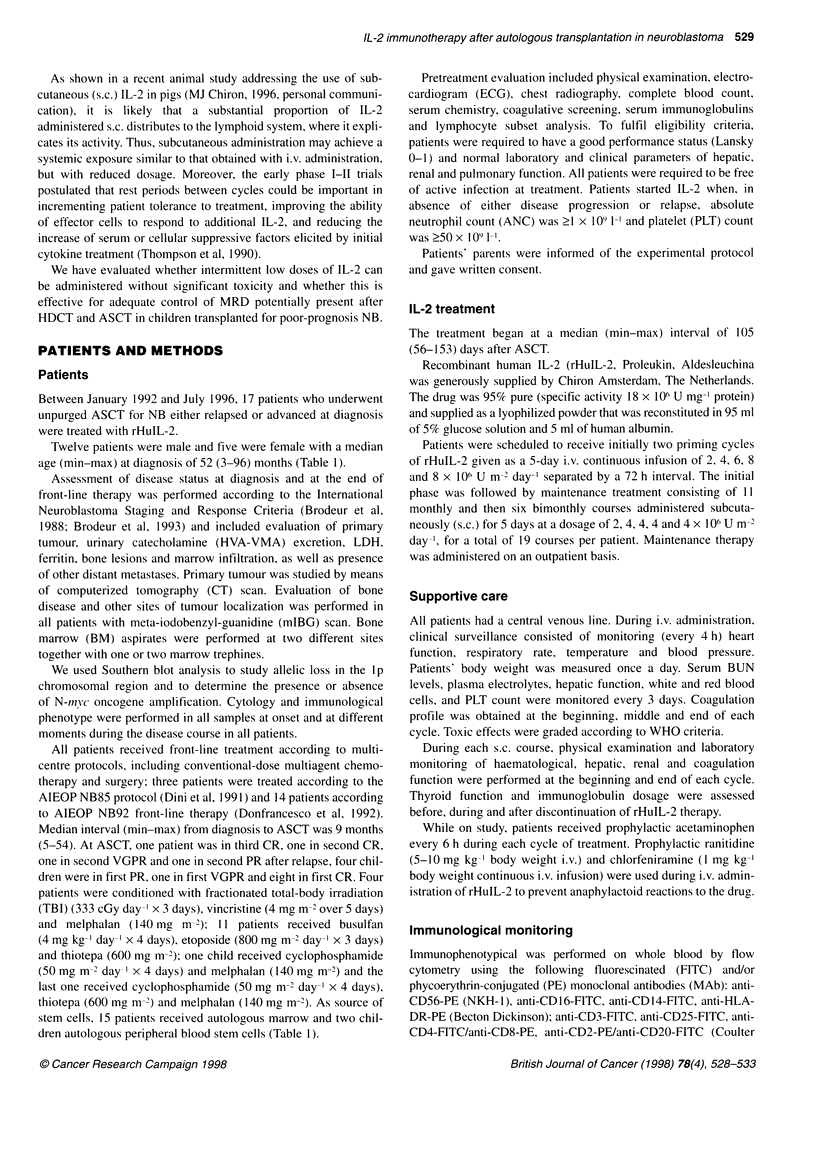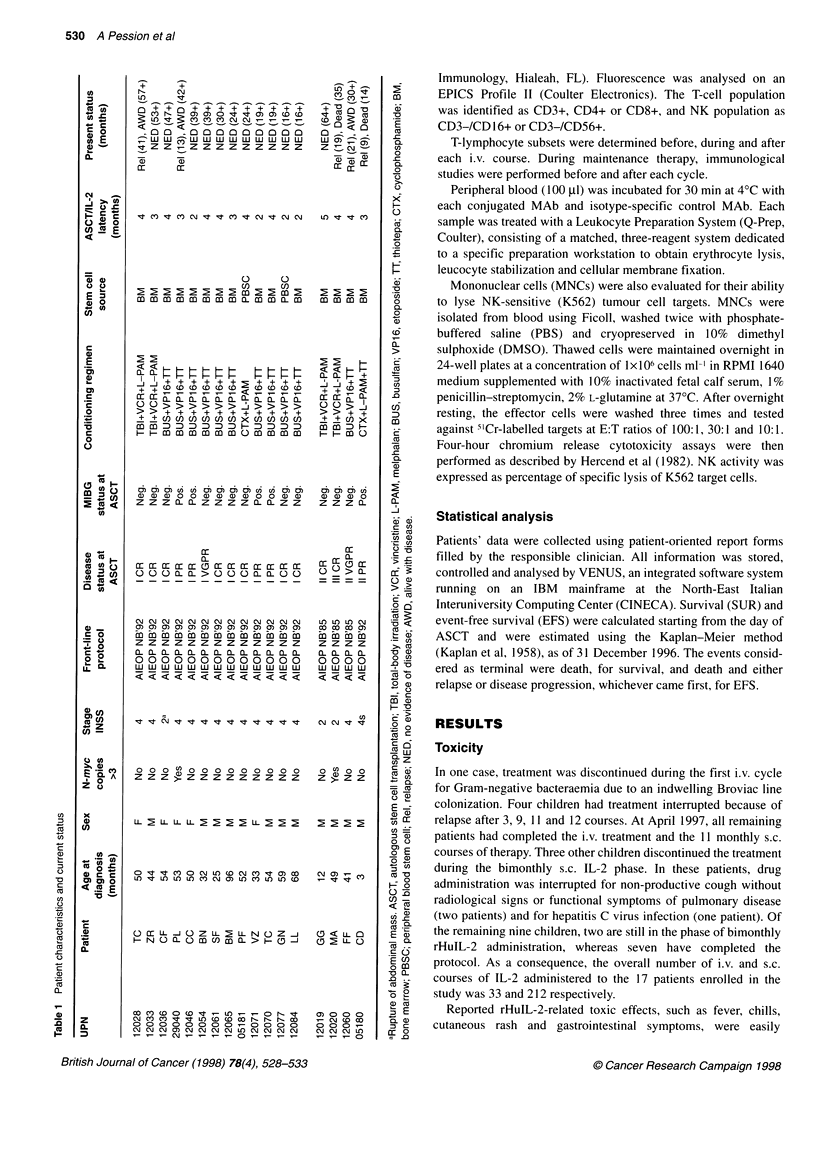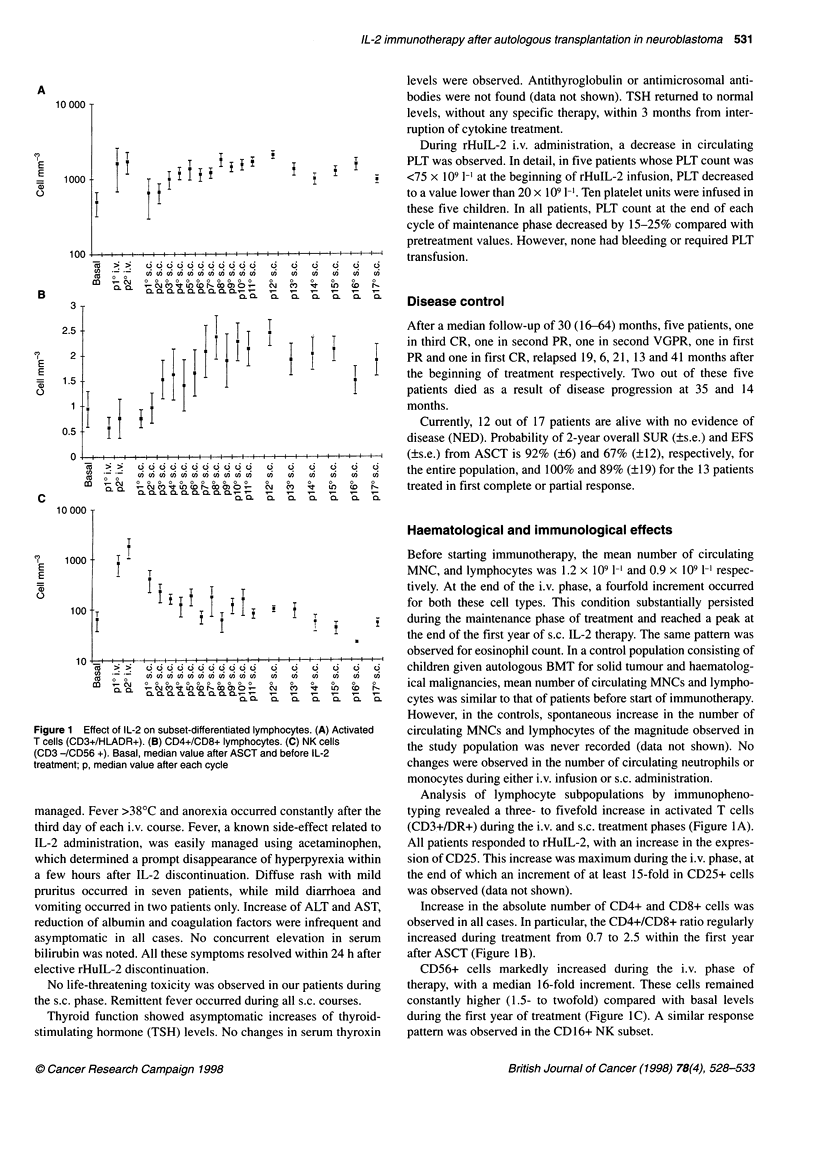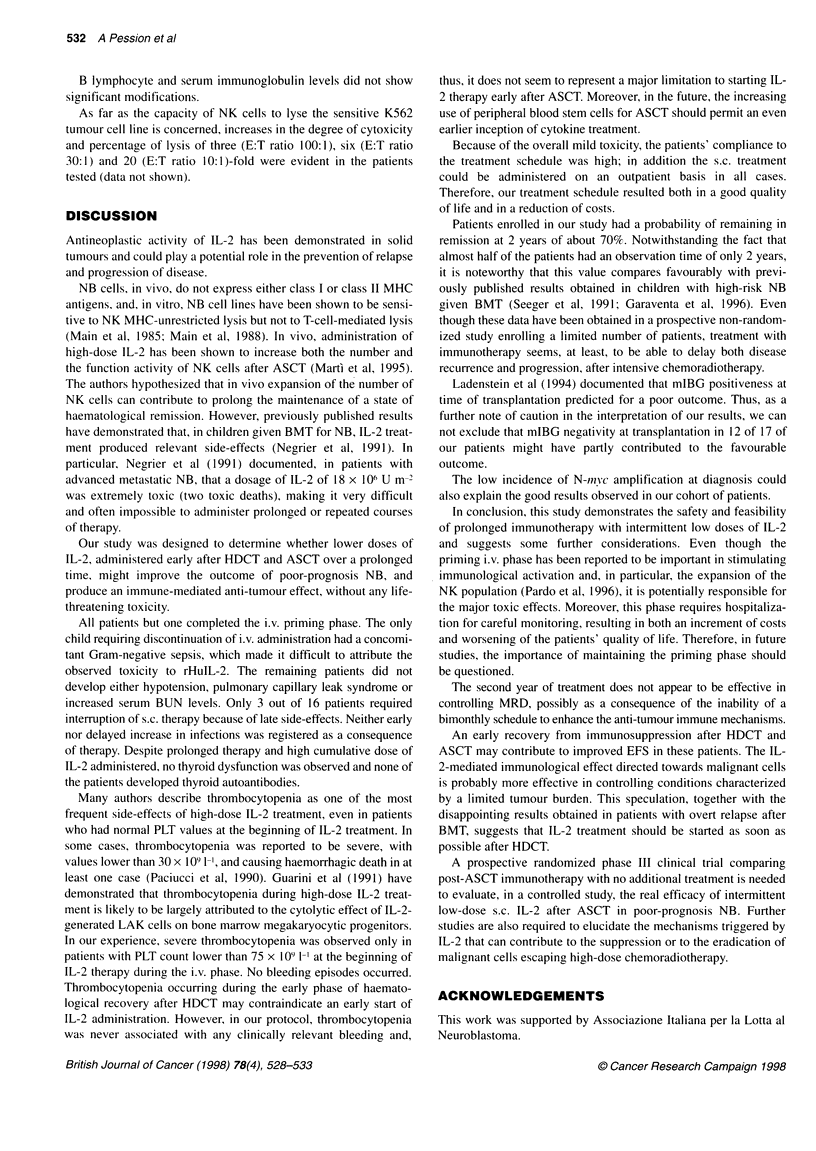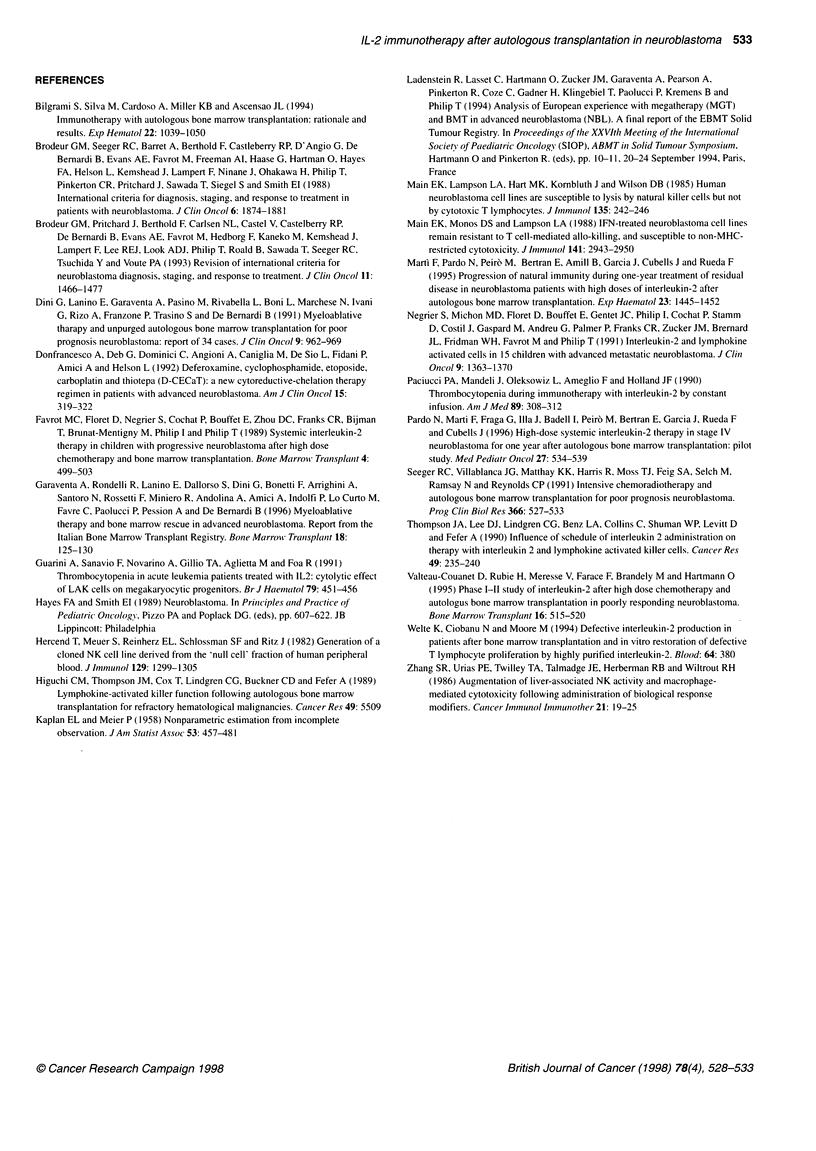# Immunotherapy with low-dose recombinant interleukin 2 after high-dose chemotherapy and autologous stem cell transplantation in neuroblastoma.

**DOI:** 10.1038/bjc.1998.527

**Published:** 1998-08

**Authors:** A. Pession, A. Prete, F. Locatelli, S. Pierinelli, A. L. Pession, R. Maccario, E. Magrini, B. De Bernardi, P. Paolucci, G. Paolucci

**Affiliations:** Clinica Pediatrica, Osp. S. Orsola-Malpighi Università di Bologna, Italy.

## Abstract

The purpose of this study was to evaluate in a phase I-II trial whether low doses of recombinant human interleukin 2 (rHuIL-2) over a prolonged period of time are safe and effective in eradicating or controlling minimal residual disease in children with neuroblastoma given high-dose chemotherapy (HDCT) and autologous stem cell transplantation (ASCT). From January 1992 to July 1996, 17 consecutive patients, with either stage IV or relapsed neuroblastoma, were enrolled. Patients received rHuIL-2 after a median time interval (min-max) of 105 days (56-153) after HDCT and ASCT. The protocol consisted of 2 'priming' courses of rHuIL-2 at escalating doses administered intravenously at 72-h intervals, followed by 'maintenance' with 11 monthly and six bimonthly boosting 5-day courses administered subcutaneously on an outpatient basis. At April 1997, 7 out of the 17 patients had completed the treatment schedule, four had discontinued treatment because of toxicity and four because of relapse; the remaining two patients are still on treatment, having completed 15 courses. Expansion of T lymphocytes, together with an increase in both natural killer cells and in activated T lymphocytes was evidenced. After a median (min-max) follow-up time of 30 (16-64) months, 12 out of 17 patients are alive and well. Two patients relapsed and died 14 and 35 months after transplant. Three patients are alive after having relapsed at 41, 21 and 13 months. The actuarial 2-year event-free survival and overall survival are 67% and 92% respectively. Intermittent administration of low doses of rHuIL-2 given for a long period of time is well tolerated and seems capable of controlling minimal residual disease after HDCT and ASCT in children with high-risk neuroblastoma.


British Journal of Cancer (1998) 78(4), 528–533
© 1998 Cancer Research Campaign

### Immunotherapy with low-dose recombinant interleukin 2 after high-dose chemotherapy and autologous stem cell transplantation in neuroblastoma

**A Pession[1], A Prete[1], F Locatelli[2], S Pierinelli[1], AL Pession[3], R Maccario[2], E Magrini[1], B De Bernardi[4], P Paolucci[5] and G Paolucci[1]**

[1]Clinica Pediatrica, Osp. S. Orsola-Malpighi Università di Bologna Via Massarenti, 11-40138 Bologna, Italy; [2]Clinica Pediatrica, IRCCS Policlinico San Matteo Università di Pavia P. le Golgi, 2-27100 Pavia, Italy; [3]Dipartimento Patologia Sperimentale, Università di Bologna Via San Giacomo 15-40100 Bologna Italy; [4]Istituto Giannina Gaslini, Via 5 Maggio, 39-16148 Genova Quarto, Italy; [5]IRCCS Casa Sollievo della Sofferenza, – 71013 S. Giovanni Rotondo, Italy

**Summary** The purpose of this study was to evaluate in a phase I–II trial whether low doses of recombinant human interleukin 2 (rHuIL-2) over a prolonged period of time are safe and effective in eradicating or controlling minimal residual disease in children with neuroblastoma given high-dose chemotherapy (HDCT) and autologous stem cell transplantation (ASCT). From January 1992 to July 1996, 17 consecutive patients, with either stage IV or relapsed neuroblastoma, were enrolled. Patients received rHuIL-2 after a median time interval (min–max) of 105 days (56–153) after HDCT and ASCT. The protocol consisted of 2 'priming' courses of rHuIL-2 at escalating doses administered intravenously at 72-h intervals, followed by 'maintenance' with 11 monthly and six bimonthly boosting 5-day courses administered subcutaneously on an outpatient basis. At April 1997, 7 out of the 17 patients had completed the treatment schedule, four had discontinued treatment because of toxicity and four because of relapse; the remaining two patients are still on treatment, having completed 15 courses. Expansion of T lymphocytes, together with an increase in both natural killer cells and in activated T lymphocytes was evidenced. After a median (min–max) follow-up time of 30 (16–64) months, 12 out of 17 patients are alive and well. Two patients relapsed and died 14 and 35 months after transplant. Three patients are alive after having relapsed at 41, 21 and 13 months. The actuarial 2-year event-free survival and overall survival are 67% and 92% respectively. Intermittent administration of low doses of rHuIL-2 given for a long period of time is well tolerated and seems capable of controlling minimal residual disease after HDCT and ASCT in children with high-risk neuroblastoma.

**Keywords:** neuroblastoma; high-dose chemotherapy; interleukin 2; immunotherapy

Disseminated neuroblastoma (NB) in children over 1 year of age at diagnosis remains one of the major challenges in paediatric oncology. In the 1970s, only 10% of patients with stage IV NB treated with conventional multimodality approaches (Hayes et al, 1989) were alive at 3 years. The introduction of intensive induction treatments followed by high-dose chemotherapy (HDCT) and autologous stem cell transplantation (ASCT) has improved the outcome, suppressing regrowth of neoplastic cells, but still failing to eradicate minimal residual disease (MRD); long-term survival is below 30% in most recent reports (Garaventa et al, 1996). In these patients, most relapses occurred within 12 months after transplantation, even though, in some cases, a clinical and radiological complete remission (CR) had been achieved.

Experimental and clinical data demonstrate that a regenerating immune system is functionally and structurally inadequate to control MRD post ASCT. For 6 months or longer, transplanted patients show an impaired immunological response and undetectable interleukin 2 (IL-2) serum levels (Bilgrami et al, 1994; Welte et al, 1994).

*Received 18 June 1997*
*Revised 19 January 1998*
*Accepted 23 January 1998*

*Correspondence to:* A Pession, Clinica Pediatrica Università di Bologna Policlinico S. Orsola Malpighi, Via Massarenti, 11 40138 Bologna, Italy

Addition of IL-2 to peripheral blood lymphocytes (PBL) from patients after ASCT markedly increases the in vitro cytotoxic activity of these cells against long-term cultured tumour targets (Higuchi et al, 1989). After IL-2 stimulation, natural killer (NK) cells (CD3–/CD16+, CD3–/CD56+) are the earliest lymphocytes to reappear after ASCT. These cells show LAK activity and major histocompatibility complex (MHC) unrestricted lysis of tumour cells. Furthermore, IL-2 enhances the anti-tumour effect of macrophages through induction of cytokines with antineoplastic activity, such as α-tumour necrosis factor (α-TNF) and γ-interferon (γ-IFN) (Zhang et al, 1986). NB is a non-immunogenic tumour, whose derived cell lines have been demonstrated to be sensitive to NK lysis (Main et al, 1985). As a consequence, great interest has arisen around the possibility that IL-2 after ASCT could potentially lead to prolonged remission or even cure in poor-prognosis NB (Favrot et al, 1989).

Phase I–II trials conducted on children with NB have demonstrated that $18 \times 10^6$ U m$^{-2}$ day$^{-1}$ represent the maximum-tolerated dose (MTD) of IL-2 in non-grafted children (D Valteau-Couanet, personal unpublished data) and $12 \times 10^6$ U m$^{-2}$ day$^{-1}$ in patients given BMT (Valteau-Couanet et al, 1995) Fever, vomiting, diarrhoea, hypertension, neurological symptoms together with jaundice, increased serum BUN levels and weight gain were frequent and sometimes life-threatening complications requiring discontinuation of treatment.

As shown in a recent animal study addressing the use of subcutaneous (s.c.) IL-2 in pigs (MJ Chiron, 1996, personal communication), it is likely that a substantial proportion of IL-2 administered s.c. distributes to the lymphoid system, where it explicates its activity. Thus, subcutaneous administration may achieve a systemic exposure similar to that obtained with i.v. administration, but with reduced dosage. Moreover, the early phase I–II trials postulated that rest periods between cycles could be important in incrementing patient tolerance to treatment, improving the ability of effector cells to respond to additional IL-2, and reducing the increase of serum or cellular suppressive factors elicited by initial cytokine treatment (Thompson et al, 1990).

We have evaluated whether intermittent low doses of IL-2 can be administered without significant toxicity and whether this is effective for adequate control of MRD potentially present after HDCT and ASCT in children transplanted for poor-prognosis NB.

### PATIENTS AND METHODS

### Patients

Between January 1992 and July 1996, 17 patients who underwent unpurged ASCT for NB either relapsed or advanced at diagnosis were treated with rHuIL-2.

Twelve patients were male and five were female with a median age (min–max) at diagnosis of 52 (3–96) months (Table 1).

Assessment of disease status at diagnosis and at the end of front-line therapy was performed according to the International Neuroblastoma Staging and Response Criteria (Brodeur et al, 1988; Brodeur et al, 1993) and included evaluation of primary tumour, urinary catecholamine (HVA-VMA) excretion, LDH, ferritin, bone lesions and marrow infiltration, as well as presence of other distant metastases. Primary tumour was studied by means of computerized tomography (CT) scan. Evaluation of bone disease and other sites of tumour localization was performed in all patients with meta-iodobenzyl-guanidine (mIBG) scan. Bone marrow (BM) aspirates were performed at two different sites together with one or two marrow trephines.

We used Southern blot analysis to study allelic loss in the 1p chromosomal region and to determine the presence or absence of N-*myc* oncogene amplification. Cytology and immunological phenotype were performed in all samples at onset and at different moments during the disease course in all patients.

All patients received front-line treatment according to multicentre protocols, including conventional-dose multiagent chemotherapy and surgery; three patients were treated according to the AIEOP NB85 protocol (Dini et al, 1991) and 14 patients according to AIEOP NB92 front-line therapy (Donfrancesco et al, 1992). Median interval (min–max) from diagnosis to ASCT was 9 months (5–54). At ASCT, one patient was in third CR, one in second CR, one in second VGPR and one in second PR after relapse, four children were in first PR, one in first VGPR and eight in first CR. Four patients were conditioned with fractionated total-body irradiation (TBI) (333 cGy day$^{-1}$ × 3 days), vincristine (4 mg m$^{-2}$ over 5 days) and melphalan (140 mg m$^{-2}$); 11 patients received busulfan (4 mg kg$^{-1}$ day$^{-1}$ × 4 days), etoposide (800 mg m$^{-2}$ day$^{-1}$ × 3 days) and thiotepa (600 mg m$^{-2}$); one child received cyclophosphamide (50 mg m$^{-2}$ day$^{-1}$ × 4 days) and melphalan (140 mg m$^{-2}$) and the last one received cyclophosphamide (50 mg m$^{-2}$ day$^{-1}$ × 4 days), thiotepa (600 mg m$^{-2}$) and melphalan (140 mg m$^{-2}$). As source of stem cells, 15 patients received autologous marrow and two children autologous peripheral blood stem cells (Table 1).

Pretreatment evaluation included physical examination, electrocardiogram (ECG), chest radiography, complete blood count, serum chemistry, coagulative screening, serum immunoglobulins and lymphocyte subset analysis. To fulfil eligibility criteria, patients were required to have a good performance status (Lansky 0–1) and normal laboratory and clinical parameters of hepatic, renal and pulmonary function. All patients were required to be free of active infection at treatment. Patients started IL-2 when, in absence of either disease progression or relapse, absolute neutrophil count (ANC) was ≥1 × 10$^{9}$ l$^{-1}$ and platelet (PLT) count was ≥50 × 10$^{9}$ l$^{-1}$.

Patients' parents were informed of the experimental protocol and gave written consent.

### IL-2 treatment

The treatment began at a median (min–max) interval of 105 (56–153) days after ASCT.

Recombinant human IL-2 (rHuIL-2, Proleukin, Aldesleuchina was generously supplied by Chiron Amsterdam, The Netherlands. The drug was 95% pure (specific activity 18 × 10$^{6}$ U mg$^{-1}$ protein) and supplied as a lyophilized powder that was reconstituted in 95 ml of 5% glucose solution and 5 ml of human albumin.

Patients were scheduled to receive initially two priming cycles of rHuIL-2 given as a 5-day i.v. continuous infusion of 2, 4, 6, 8 and 8 × 10$^{6}$ U m$^{-2}$ day$^{-1}$ separated by a 72 h interval. The initial phase was followed by maintenance treatment consisting of 11 monthly and then six bimonthly courses administered subcutaneously (s.c.) for 5 days at a dosage of 2, 4, 4, 4 and 4 × 10$^{6}$ U m$^{-2}$ day$^{-1}$, for a total of 19 courses per patient. Maintenance therapy was administered on an outpatient basis.

### Supportive care

All patients had a central venous line. During i.v. administration, clinical surveillance consisted of monitoring (every 4 h) heart function, respiratory rate, temperature and blood pressure. Patients' body weight was measured once a day. Serum BUN levels, plasma electrolytes, hepatic function, white and red blood cells, and PLT count were monitored every 3 days. Coagulation profile was obtained at the beginning, middle and end of each cycle. Toxic effects were graded according to WHO criteria.

During each s.c. course, physical examination and laboratory monitoring of haematological, hepatic, renal and coagulation function were performed at the beginning and end of each cycle. Thyroid function and immunoglobulin dosage were assessed before, during and after discontinuation of rHuIL-2 therapy.

While on study, patients received prophylactic acetaminophen every 6 h during each cycle of treatment. Prophylactic ranitidine (5–10 mg kg$^{-1}$ body weight i.v.) and chlorfeniramine (1 mg kg$^{-1}$ body weight continuous i.v. infusion) were used during i.v. administration of rHuIL-2 to prevent anaphylactoid reactions to the drug.

### Immunological monitoring

Immunophenotypical was performed on whole blood by flow cytometry using the following fluorescinated (FITC) and/or phycoerythrin-conjugated (PE) monoclonal antibodies (MAb): anti-CD56-PE (NKH-1), anti-CD16-FITC, anti-CD14-FITC, anti-HLA-DR-PE (Becton Dickinson); anti-CD3-FITC, anti-CD25-FITC, anti-CD4-FITC/anti-CD8-PE, anti-CD2-PE/anti-CD20-FITC (Coulter

© Cancer Research Campaign 1998

**Table 1** Patient characteristics and current status

| UPN | Patient | Age at diagnosis (months) | Sex | *N-myc* copies >3 | Stage INSS | Front-line protocol | Disease status at ASCT | MIBG status at ASCT | Conditioning regimen | Stem cell source | ASCT/IL-2 latency (months) | Present status (months) |
|---|---|---|---|---|---|---|---|---|---|---|---|---|
| 12028 | TC | 50 | F | No | 4 | AIEOP NB'92 | I CR | Neg. | TBI+VCR+L–PAM | BM | 4 | Rel (41), AWD (57+) |
| 12033 | ZR | 44 | M | No | 4 | AIEOP NB'92 | I CR | Neg. | TBI+VCR+L–PAM | BM | 3 | NED (53+) |
| 12036 | CF | 54 | F | No | 2[a] | AIEOP NB'92 | I CR | Neg. | BUS+VP16+TT | BM | 4 | NED (47+) |
| 29040 | PL | 53 | F | Yes | 4 | AIEOP NB'92 | I PR | Pos. | BUS+VP16+TT | BM | 3 | Rel (13), AWD (42+) |
| 12046 | CC | 50 | F | No | 4 | AIEOP NB'92 | I PR | Pos. | BUS+VP16+TT | BM | 2 | NED (39+) |
| 12054 | BN | 32 | M | No | 4 | AIEOP NB'92 | I VGPR | Neg. | BUS+VP16+TT | BM | 4 | NED (39+) |
| 12061 | SF | 25 | M | No | 4 | AIEOP NB'92 | I CR | Neg. | BUS+VP16+TT | BM | 4 | NED (30+) |
| 12065 | BM | 96 | M | No | 4 | AIEOP NB'92 | I CR | Neg. | BUS+VP16+TT | BM | 3 | NED (24+) |
| 05181 | PF | 52 | M | No | 4 | AIEOP NB'92 | I CR | Neg. | CTX+L-PAM | PBSC | 4 | NED (24+) |
| 12071 | VZ | 33 | F | No | 4 | AIEOP NB'92 | I PR | Pos. | BUS+VP16+TT | BM | 2 | NED (19+) |
| 12070 | TC | 54 | M | No | 4 | AIEOP NB'92 | I PR | Pos. | BUS+VP16+TT | BM | 4 | NED (19+) |
| 12077 | GN | 59 | M | No | 4 | AIEOP NB'92 | I CR | Neg. | BUS+VP16+TT | PBSC | 2 | NED (16+) |
| 12084 | LL | 68 | M | No | 4 | AIEOP NB'92 | I CR | Neg. | BUS+VP16+TT | BM | 2 | NED (16+) |
| 12019 | GG | 12 | M | No | 2 | AIEOP NB'85 | II CR | Neg. | TBI+VCR+L-PAM | BM | 5 | NED (64+) |
| 12020 | MA | 49 | M | Yes | 2 | AIEOP NB'85 | III CR | Neg. | TBI+VCR+L-PAM | BM | 4 | Rel (19), Dead (35) |
| 12060 | FF | 41 | M | No | 4 | AIEOP NB'85 | II VGPR | Neg. | BUS+VP16+TT | BM | 4 | Rel (21), AWD (30+) |
| 05180 | CD | 3 | M | No | 4s | AIEOP NB'92 | II PR | Pos. | CTX+L–PAM+TT | BM | 3 | Rel (9), Dead (14) |

[a]Rupture of abdominal mass. ASCT, autologous stem cell transplantation; TBI, total-body irradiation; VCR, vincristine; L-PAM, melphalan; BUS, busulfan; VP16, etoposide; TT, thiotepa; CTX, cyclophosphamide; BM, bone marrow; PBSC; peripheral blood stem cell; Rel, relapse; NED, no evidence of disease; AWD, alive with disease.

Immunology, Hialeah, FL). Fluorescence was analysed on an EPICS Profile II (Coulter Electronics). The T-cell population was identified as CD3+, CD4+ or CD8+, and NK population as CD3–/CD16+ or CD3–/CD56+.

T-lymphocyte subsets were determined before, during and after each i.v. course. During maintenance therapy, immunological studies were performed before and after each cycle.

Peripheral blood (100 μl) was incubated for 30 min at 4°C with each conjugated MAb and isotype-specific control MAb. Each sample was treated with a Leukocyte Preparation System (Q-Prep, Coulter), consisting of a matched, three-reagent system dedicated to a specific preparation workstation to obtain erythrocyte lysis, leucocyte stabilization and cellular membrane fixation.

Mononuclear cells (MNCs) were also evaluated for their ability to lyse NK-sensitive (K562) tumour cell targets. MNCs were isolated from blood using Ficoll, washed twice with phosphate-buffered saline (PBS) and cryopreserved in 10% dimethyl sulphoxide (DMSO). Thawed cells were maintained overnight in 24-well plates at a concentration of $1 \times 10^6$ cells $ml^{-1}$ in RPMI 1640 medium supplemented with 10% inactivated fetal calf serum, 1% penicillin–streptomycin, 2% L-glutamine at 37°C. After overnight resting, the effector cells were washed three times and tested against $^{51}Cr$-labelled targets at E:T ratios of 100:1, 30:1 and 10:1. Four-hour chromium release cytotoxicity assays were then performed as described by Hercend et al (1982). NK activity was expressed as percentage of specific lysis of K562 target cells.

### Statistical analysis

Patients' data were collected using patient-oriented report forms filled by the responsible clinician. All information was stored, controlled and analysed by VENUS, an integrated software system running on an IBM mainframe at the North-East Italian Interuniversity Computing Center (CINECA). Survival (SUR) and event-free survival (EFS) were calculated starting from the day of ASCT and were estimated using the Kaplan–Meier method (Kaplan et al, 1958), as of 31 December 1996. The events considered as terminal were death, for survival, and death and either relapse or disease progression, whichever came first, for EFS.

### RESULTS

### Toxicity

In one case, treatment was discontinued during the first i.v. cycle for Gram-negative bacteraemia due to an indwelling Broviac line colonization. Four children had treatment interrupted because of relapse after 3, 9, 11 and 12 courses. At April 1997, all remaining patients had completed the i.v. treatment and the 11 monthly s.c. courses of therapy. Three other children discontinued the treatment during the bimonthly s.c. IL-2 phase. In these patients, drug administration was interrupted for non-productive cough without radiological signs or functional symptoms of pulmonary disease (two patients) and for hepatitis C virus infection (one patient). Of the remaining nine children, two are still in the phase of bimonthly rHuIL-2 administration, whereas seven have completed the protocol. As a consequence, the overall number of i.v. and s.c. courses of IL-2 administered to the 17 patients enrolled in the study was 33 and 212 respectively.

Reported rHuIL-2-related toxic effects, such as fever, chills, cutaneous rash and gastrointestinal symptoms, were easily

© Cancer Research Campaign 1998

**Figure 1** Effect of IL-2 on subset-differentiated lymphocytes. (**A**) Activated T cells (CD3+/HLADR+). (**B**) CD4+/CD8+ lymphocytes. (**C**) NK cells (CD3 –/CD56 +). Basal, median value after ASCT and before IL-2 treatment; p, median value after each cycle managed. Fever >38°C and anorexia occurred constantly after the third day of each i.v. course. Fever, a known side-effect related to IL-2 administration, was easily managed using acetaminophen, which determined a prompt disappearance of hyperpyrexia within a few hours after IL-2 discontinuation. Diffuse rash with mild pruritus occurred in seven patients, while mild diarrhoea and vomiting occurred in two patients only. Increase of ALT and AST, reduction of albumin and coagulation factors were infrequent and asymptomatic in all cases. No concurrent elevation in serum bilirubin was noted. All these symptoms resolved within 24 h after elective rHuIL-2 discontinuation.

No life-threatening toxicity was observed in our patients during the s.c. phase. Remittent fever occurred during all s.c. courses.

Thyroid function showed asymptomatic increases of thyroid-stimulating hormone (TSH) levels. No changes in serum thyroxin levels were observed. Antithyroglobulin or antimicrosomal antibodies were not found (data not shown). TSH returned to normal levels, without any specific therapy, within 3 months from interruption of cytokine treatment.

During rHuIL-2 i.v. administration, a decrease in circulating PLT was observed. In detail, in five patients whose PLT count was $<75 \times 10^9\ l^{-1}$ at the beginning of rHuIL-2 infusion, PLT decreased to a value lower than $20 \times 10^9\ l^{-1}$. Ten platelet units were infused in these five children. In all patients, PLT count at the end of each cycle of maintenance phase decreased by 15–25% compared with pretreatment values. However, none had bleeding or required PLT transfusion.

### Disease control

After a median follow-up of 30 (16–64) months, five patients, one in third CR, one in second PR, one in second VGPR, one in first PR and one in first CR, relapsed 19, 6, 21, 13 and 41 months after the beginning of treatment respectively. Two out of these five patients died as a result of disease progression at 35 and 14 months.

Currently, 12 out of 17 patients are alive with no evidence of disease (NED). Probability of 2-year overall SUR (±s.e.) and EFS (±s.e.) from ASCT is 92% (±6) and 67% (±12), respectively, for the entire population, and 100% and 89% (±19) for the 13 patients treated in first complete or partial response.

### Haematological and immunological effects

Before starting immunotherapy, the mean number of circulating MNC, and lymphocytes was $1.2 \times 10^9\ l^{-1}$ and $0.9 \times 10^9\ l^{-1}$ respectively. At the end of the i.v. phase, a fourfold increment occurred for both these cell types. This condition substantially persisted during the maintenance phase of treatment and reached a peak at the end of the first year of s.c. IL-2 therapy. The same pattern was observed for eosinophil count. In a control population consisting of children given autologous BMT for solid tumour and haematological malignancies, mean number of circulating MNCs and lymphocytes was similar to that of patients before start of immunotherapy. However, in the controls, spontaneous increase in the number of circulating MNCs and lymphocytes of the magnitude observed in the study population was never recorded (data not shown). No changes were observed in the number of circulating neutrophils or monocytes during either i.v. infusion or s.c. administration.

Analysis of lymphocyte subpopulations by immunophenotyping revealed a three- to fivefold increase in activated T cells (CD3+/DR+) during the i.v. and s.c. treatment phases (Figure 1A). All patients responded to rHuIL-2, with an increase in the expression of CD25. This increase was maximum during the i.v. phase, at the end of which an increment of at least 15-fold in CD25+ cells was observed (data not shown).

Increase in the absolute number of CD4+ and CD8+ cells was observed in all cases. In particular, the CD4+/CD8+ ratio regularly increased during treatment from 0.7 to 2.5 within the first year after ASCT (Figure 1B).

CD56+ cells markedly increased during the i.v. phase of therapy, with a median 16-fold increment. These cells remained constantly higher (1.5- to twofold) compared with basal levels during the first year of treatment (Figure 1C). A similar response pattern was observed in the CD16+ NK subset.

© Cancer Research Campaign 1998

B lymphocyte and serum immunoglobulin levels did not show significant modifications.

As far as the capacity of NK cells to lyse the sensitive K562 tumour cell line is concerned, increases in the degree of cytoxicity and percentage of lysis of three (E:T ratio 100:1), six (E:T ratio 30:1) and 20 (E:T ratio 10:1)-fold were evident in the patients tested (data not shown).

### DISCUSSION

Antineoplastic activity of IL-2 has been demonstrated in solid tumours and could play a potential role in the prevention of relapse and progression of disease.

NB cells, in vivo, do not express either class I or class II MHC antigens, and, in vitro, NB cell lines have been shown to be sensitive to NK MHC-unrestricted lysis but not to T-cell-mediated lysis (Main et al, 1985; Main et al, 1988). In vivo, administration of high-dose IL-2 has been shown to increase both the number and the function activity of NK cells after ASCT (Martì et al, 1995). The authors hypothesized that in vivo expansion of the number of NK cells can contribute to prolong the maintenance of a state of haematological remission. However, previously published results have demonstrated that, in children given BMT for NB, IL-2 treatment produced relevant side-effects (Negrier et al, 1991). In particular, Negrier et al (1991) documented, in patients with advanced metastatic NB, that a dosage of IL-2 of $18 \times 10^6$ U $m^{-2}$ was extremely toxic (two toxic deaths), making it very difficult and often impossible to administer prolonged or repeated courses of therapy.

Our study was designed to determine whether lower doses of IL-2, administered early after HDCT and ASCT over a prolonged time, might improve the outcome of poor-prognosis NB, and produce an immune-mediated anti-tumour effect, without any life-threatening toxicity.

All patients but one completed the i.v. priming phase. The only child requiring discontinuation of i.v. administration had a concomitant Gram-negative sepsis, which made it difficult to attribute the observed toxicity to rHuIL-2. The remaining patients did not develop either hypotension, pulmonary capillary leak syndrome or increased serum BUN levels. Only 3 out of 16 patients required interruption of s.c. therapy because of late side-effects. Neither early nor delayed increase in infections was registered as a consequence of therapy. Despite prolonged therapy and high cumulative dose of IL-2 administered, no thyroid dysfunction was observed and none of the patients developed thyroid autoantibodies.

Many authors describe thrombocytopenia as one of the most frequent side-effects of high-dose IL-2 treatment, even in patients who had normal PLT values at the beginning of IL-2 treatment. In some cases, thrombocytopenia was reported to be severe, with values lower than $30 \times 10^9$ $l^{-1}$, and causing haemorrhagic death in at least one case (Paciucci et al, 1990). Guarini et al (1991) have demonstrated that thrombocytopenia during high-dose IL-2 treatment is likely to be largely attributed to the cytolytic effect of IL-2-generated LAK cells on bone marrow megakaryocytic progenitors. In our experience, severe thrombocytopenia was observed only in patients with PLT count lower than $75 \times 10^9$ $l^{-1}$ at the beginning of IL-2 therapy during the i.v. phase. No bleeding episodes occurred. Thrombocytopenia occurring during the early phase of haematological recovery after HDCT may contraindicate an early start of IL-2 administration. However, in our protocol, thrombocytopenia was never associated with any clinically relevant bleeding and, thus, it does not seem to represent a major limitation to starting IL-2 therapy early after ASCT. Moreover, in the future, the increasing use of peripheral blood stem cells for ASCT should permit an even earlier inception of cytokine treatment.

Because of the overall mild toxicity, the patients' compliance to the treatment schedule was high; in addition the s.c. treatment could be administered on an outpatient basis in all cases. Therefore, our treatment schedule resulted both in a good quality of life and in a reduction of costs.

Patients enrolled in our study had a probability of remaining in remission at 2 years of about 70%. Notwithstanding the fact that almost half of the patients had an observation time of only 2 years, it is noteworthy that this value compares favourably with previously published results obtained in children with high-risk NB given BMT (Seeger et al, 1991; Garaventa et al, 1996). Even though these data have been obtained in a prospective non-randomized study enrolling a limited number of patients, treatment with immunotherapy seems, at least, to be able to delay both disease recurrence and progression, after intensive chemoradiotherapy.

Ladenstein et al (1994) documented that mIBG positiveness at time of transplantation predicted for a poor outcome. Thus, as a further note of caution in the interpretation of our results, we can not exclude that mIBG negativity at transplantation in 12 of 17 of our patients might have partly contributed to the favourable outcome.

The low incidence of N-*myc* amplification at diagnosis could also explain the good results observed in our cohort of patients.

In conclusion, this study demonstrates the safety and feasibility of prolonged immunotherapy with intermittent low doses of IL-2 and suggests some further considerations. Even though the priming i.v. phase has been reported to be important in stimulating immunological activation and, in particular, the expansion of the NK population (Pardo et al, 1996), it is potentially responsible for the major toxic effects. Moreover, this phase requires hospitalization for careful monitoring, resulting in both an increment of costs and worsening of the patients' quality of life. Therefore, in future studies, the importance of maintaining the priming phase should be questioned.

The second year of treatment does not appear to be effective in controlling MRD, possibly as a consequence of the inability of a bimonthly schedule to enhance the anti-tumour immune mechanisms.

An early recovery from immunosuppression after HDCT and ASCT may contribute to improved EFS in these patients. The IL-2-mediated immunological effect directed towards malignant cells is probably more effective in controlling conditions characterized by a limited tumour burden. This speculation, together with the disappointing results obtained in patients with overt relapse after BMT, suggests that IL-2 treatment should be started as soon as possible after HDCT.

A prospective randomized phase III clinical trial comparing post-ASCT immunotherapy with no additional treatment is needed to evaluate, in a controlled study, the real efficacy of intermittent low-dose s.c. IL-2 after ASCT in poor-prognosis NB. Further studies are also required to elucidate the mechanisms triggered by IL-2 that can contribute to the suppression or to the eradication of malignant cells escaping high-dose chemoradiotherapy.

### ACKNOWLEDGEMENTS

This work was supported by Associazione Italiana per la Lotta al Neuroblastoma.

© Cancer Research Campaign 1998

### REFERENCES

Bilgrami S, Silva M, Cardoso A, Miller KB and Ascensao JL (1994) Immunotherapy with autologous bone marrow transplantation: rationale and results. *Exp Hematol* **22**: 1039–1050

Brodeur GM, Seeger RC, Barret A, Berthold F, Castleberry RP, D'Angio G, De Bernardi B, Evans AE, Favrot M, Freeman AI, Haase G, Hartman O, Hayes FA, Helson L, Kemshead J, Lampert F, Ninane J, Ohakawa H, Philip T, Pinkerton CR, Pritchard J, Sawada T, Siegel S and Smith EI (1988) International criteria for diagnosis, staging, and response to treatment in patients with neuroblastoma. *J Clin Oncol* **6**: 1874–1881

Brodeur GM, Pritchard J, Berthold F, Carlsen NL, Castel V, Castelberry RP, De Bernardi B, Evans AE, Favrot M, Hedborg F, Kaneko M, Kemshead J, Lampert F, Lee REJ, Look ADJ, Philip T, Roald B, Sawada T, Seeger RC, Tsuchida Y and Voute PA (1993) Revision of international criteria for neuroblastoma diagnosis, staging, and response to treatment. *J Clin Oncol* **11**: 1466–1477

Dini G, Lanino E, Garaventa A, Pasino M, Rivabella L, Boni L, Marchese N, Ivani G, Rizo A, Franzone P, Trasino S and De Bernardi B (1991) Myeloablative tharapy and unpurged autologous bone marrow transplantation for poor prognosis neuroblastoma: report of 34 cases. *J Clin Oncol* **9**: 962–969

Donfrancesco A, Deb G, Dominici C, Angioni A, Caniglia M, De Sio L, Fidani P, Amici A and Helson L (1992) Deferoxamine, cyclophosphamide, etoposide, carboplatin and thiotepa (D-CECaT): a new cytoreductive-chelation therapy regimen in patients with advanced neuroblastoma. *Am J Clin Oncol* **15**: 319–322

Favrot MC, Floret D, Negrier S, Cochat P, Bouffet E, Zhou DC, Franks CR, Bijman T, Brunat-Mentigny M, Philip I and Philip T (1989) Systemic interleukin-2 therapy in children with progressive neuroblastoma after high dose chemotherapy and bone marrow transplantation. *Bone Marrow Transplant* **4**: 499–503

Garaventa A, Rondelli R, Lanino E, Dallorso S, Dini G, Bonetti F, Arrighini A, Santoro N, Rossetti F, Miniero R, Andolina A, Amici A, Indolfi P, Lo Curto M, Favre C, Paolucci P, Pession A and De Bernardi B (1996) Myeloablative therapy and bone marrow rescue in advanced neuroblastoma. Report from the Italian Bone Marrow Transplant Registry. *Bone Marrow Transplant* **18**: 125–130

Guarini A, Sanavio F, Novarino A, Gillio TA, Aglietta M and Foa R (1991) Thrombocytopenia in acute leukemia patients treated with IL2: cytolytic effect of LAK cells on megakaryocytic progenitors. *Br J Haematol* **79**: 451–456

Hayes FA and Smith EI (1989) Neuroblastoma. In *Principles and Practice of Pediatric Oncology*, Pizzo PA and Poplack DG. (eds), pp. 607–622. JB Lippincott: Philadelphia

Hercend T, Meuer S, Reinherz EL, Schlossman SF and Ritz J (1982) Generation of a cloned NK cell line derived from the 'null cell' fraction of human peripheral blood. *J Immunol* **129**: 1299–1305

Higuchi CM, Thompson JM, Cox T, Lindgren CG, Buckner CD and Fefer A (1989) Lymphokine-activated killer function following autologous bone marrow transplantation for refractory hematological malignancies. *Cancer Res* **49**: 5509

Kaplan EL and Meier P (1958) Nonparametric estimation from incomplete observation. *J Am Statist Assoc* **53**: 457–481

Ladenstein R, Lasset C, Hartmann O, Zucker JM, Garaventa A, Pearson A, Pinkerton R, Coze C, Gadner H, Klingebiel T, Paolucci P, Kremens B and Philip T (1994) Analysis of European experience with megatherapy (MGT) and BMT in advanced neuroblastoma (NBL). A final report of the EBMT Solid Tumour Registry. In *Proceedings of the XXVIth Meeting of the International Society of Paediatric Oncology* (SIOP), *ABMT in Solid Tumour Symposium*, Hartmann O and Pinkerton R. (eds), pp. 10–11, 20–24 September 1994, Paris, France

Main EK, Lampson LA, Hart MK, Kornbluth J and Wilson DB (1985) Human neuroblastoma cell lines are susceptible to lysis by natural killer cells but not by cytotoxic T lymphocytes. *J Immunol* **135**: 242–246

Main EK, Monos DS and Lampson LA (1988) IFN-treated neuroblastoma cell lines remain resistant to T cell-mediated allo-killing, and susceptible to non-MHC-restricted cytotoxicity. *J Immunol* **141**: 2943–2950

Martì F, Pardo N, Peirò M, Bertran E, Amill B, Garcia J, Cubells J and Rueda F (1995) Progression of natural immunity during one-year treatment of residual disease in neuroblastoma patients with high doses of interleukin-2 after autologous bone marrow transplantation. *Exp Haematol* **23**: 1445–1452

Negrier S, Michon MD, Floret D, Bouffet E, Gentet JC, Philip I, Cochat P, Stamm D, Costil J, Gaspard M, Andreu G, Palmer P, Franks CR, Zucker JM, Brernard JL, Fridman WH, Favrot M and Philip T (1991) Interleukin-2 and lymphokine activated cells in 15 children with advanced metastatic neuroblastoma. *J Clin Oncol* **9**: 1363–1370

Paciucci PA, Mandeli J, Oleksowiz L, Ameglio F and Holland JF (1990) Thrombocytopenia during immunotherapy with interleukin-2 by constant infusion. *Am J Med* **89**: 308–312

Pardo N, Marti F, Fraga G, Illa J, Badell I, Peirò M, Bertran E, Garcia J, Rueda F and Cubells J (1996) High-dose systemic interleukin-2 therapy in stage IV neuroblastoma for one year after autologous bone marrow transplantation: pilot study. *Med Pediatr Oncol* **27**: 534–539

Seeger RC, Villablanca JG, Matthay KK, Harris R, Moss TJ, Feig SA, Selch M, Ramsay N and Reynolds CP (1991) Intensive chemoradiotherapy and autologous bone marrow transplantation for poor prognosis neuroblastoma. *Prog Clin Biol Res* **366**: 527–533

Thompson JA, Lee DJ, Lindgren CG, Benz LA, Collins C, Shuman WP, Levitt D and Fefer A (1990) Influence of schedule of interleukin 2 administration on therapy with interleukin 2 and lymphokine activated killer cells. *Cancer Res* **49**: 235–240

Valteau-Couanet D, Rubie H, Meresse V, Farace F, Brandely M and Hartmann O (1995) Phase I–II study of interleukin-2 after high dose chemotherapy and autologus bone marrow transplantation in poorly responding neuroblastoma. *Bone Marrow Transplant* **16**: 515–520

Welte K, Ciobanu N and Moore M (1994) Defective interleukin-2 production in patients after bone marrow transplantation and in vitro restoration of defective T lymphocyte proliferation by highly purified interleukin-2. *Blood*: **64**: 380

Zhang SR, Urias PE, Twilley TA, Talmadge JE, Herberman RB and Wiltrout RH (1986) Augmentation of liver-associated NK activity and macrophage-mediated cytotoxicity following administration of biological response modifiers. *Cancer Immunol Immunother* **21**: 19–25

© Cancer Research Campaign 1998